# An adjunctive human-animal interaction intervention for veterans with PTSD: study protocol for a randomized controlled trial

**DOI:** 10.1186/s13063-019-3877-3

**Published:** 2019-12-27

**Authors:** Anouk L. Grubaugh, Ursula S. Myers, Stephanie M. Keller, Bethany C. Wangelin, Brian E. Lozano, Peter W. Tuerk

**Affiliations:** 1 Ralph H. Johnson Veterans Affairs Medical Center and Department of Psychiatry and Behavioral Sciences, Medical University of South Carolina, 109 Bee Street, Charleston, SC 29401 USA; 2 Sheila C. Johnson Center for Clinical Services, Department of Human Services, Curry School of Education, University of Virginia, 417 Emmet Street South, 400270, Charlottesville, VA 22904 USA

**Keywords:** Posttraumatic stress disorder (PTSD), Prolonged exposure (PE), Military, Veterans, Human-animal interaction (HAI), Animal-assisted therapy

## Abstract

**Background:**

Posttraumatic stress disorder (PTSD) rarely remits over time, and if left untreated, leads to significant distress, functional impairment, and increased health care costs. Fortunately, effective evidence-based treatments (EBTs) for PTSD, such as Prolonged Exposure (PE), exist. Despite their availability and efficacy, a significant number of individuals with PTSD do not initiate treatment when offered or dropout prematurely. One proposed theory suggests that the emotional-numbing symptoms of PTSD (e.g., blunted affect, apathy) can serve as a barrier to engaging in, and successfully completing, treatment; and the broad human-animal interaction (HAI) literature available suggests that HAI can potentially reduce emotional numbing related to PTSD. Accordingly, this manuscript describes an ongoing, federally funded, randomized controlled trial testing the efficacy of RESCUE, an HAI intervention, as a viable adjunctive treatment component for PE.

**Methods/design:**

The study will include 70 veterans with PTSD treated at a Southeastern Veterans Affairs Medical Center (VAMC). All participants in the trial receive up to 12 sessions of PE. Participants are randomly assigned 1:1 to (1) volunteer at a local animal shelter or (2) volunteer at a community agency of their choice as part of their in-vivo exposure exercises for PE. Outcomes will be examined via standard clinical interviews, self-report questionnaires, and thematic interviews.

**Discussion:**

It is hypothesized that participants in the HAI condition will report greater decreases in emotional-numbing symptoms and increased treatment compliance and completion rates relative to those in the community volunteer condition. If successful, RESCUE, could be easily incorporated into standard PE and broadly disseminated.

**Trial registration:**

ClinicalTrials.gov. ID: NCT03504722. Retrospectively registered on 2 May 2017.

## Background

Prolonged Exposure (PE) is considered an evidence-based treatment (EBT) for PTSD [1, 2] and has been widely disseminated throughout Department of Defense (DoD) and Veterans Affairs (VA) treatment facilities. Although up to 80% of patients demonstrate clinically meaningful improvement after completing an EBT for PTSD such as PE [1–3], a significant number of individuals do not initiate PTSD treatment or drop out of treatment prematurely (as high as 78% and 36% overall pooled dropout rates across studies) [4–7]. Given the wide-scale availability of PE and other EBTs for PTSD in VA and DoD treatment settings, and the robust effect sizes for treatment completers, there is a need for novel methods that can improve PTSD treatment engagement and decrease attrition.

One potential avenue for improving EBTs for PTSD is by targeting PTSD symptoms that may adversely impact treatment engagement early in the treatment process. Emotional numbing is part of the symptom profile of PTSD and consists of symptoms that reflect difficulties in experiencing positive emotions such as love and happiness, loss of interest in activities that were previously important or pleasurable, and feeling distant and cut off from others. In relation to PTSD and other psychiatric disorders, emotional numbing is also associated with apathy and low motivation [8, 9]. Together, these symptoms can limit an individual’s ability and willingness to engage in treatment [10, 11], particularly a treatment that encourages connecting emotionally with trauma-related material. Consistent with this, poor response to PE has been associated with blunted emotional reactivity to trauma cues early in therapy [12].

A promising mechanism for decreasing emotional numbing is through therapeutic human-animal interaction (HAI [13–15]). HAI refers to the purposeful and semistructured social pairing of a human with a domesticated animal for therapeutic purposes. A number of diverse studies have found that HAI can have positive effects on emotional and physical functioning, stress reactivity, and social functioning [16–18]; and among those with psychiatric symptoms, HAI interventions have been found to improve depression, anxiety, and PTSD [16, 19, 20]. Although promising, the HAI literature is tempered by methodological limitations, such as difficult-to-replicate study procedures, over-reliance on self-report measures, and lack of adequate controls/randomization (e.g., [18–20]).

Proactive targeting of emotional-numbing symptoms in the early stages of PTSD treatment can potentially improve treatment engagement and retention. Consistent with this rationale, we propose to test the efficacy of an HAI adjunct to PE for decreasing emotional-numbing symptoms and improving treatment engagement and outcomes. HAI is a compelling adjunct for PTSD EBTs due to its potential impact on patient-level outcomes as well as its viability for wide-scale implementation and dissemination.

### Study overview

The current study is a DoD-sponsored project (W81XWH-15-1-0087) designed to develop and pilot test the feasibility, acceptability, and efficacy of an adjunctive intervention for increasing treatment compliance with EBTs for PTSD by targeting the emotional-numbing symptoms of PTSD. RESCUE, *Recovery through Engagement with Shelter Canines, Understanding, and Exposure*, is a HAI intervention that was developed during the initial stages of the project as an adjunct to Prolonged Exposure for PTSD (PE). The innovative study design focuses on increasing PTSD treatment engagement through HAI designed to target the emotional-numbing symptoms of PTSD which are theorized to impede treatment compliance and retention. Although the current trial focuses on veterans treated at a Veterans Affairs Medical Center (VAMC), the study design and findings will have significant relevance for the broader population of individuals with PTSD across diverse treatment settings.

**Fig. 1:**
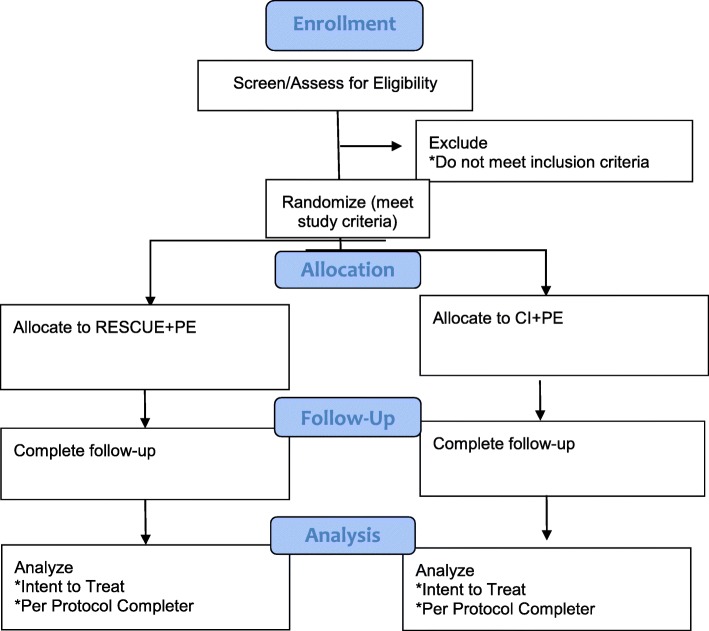
Consolidated Standards of Reporting Trials (CONSORT) Diagram

Feasibility, acceptability, and initial efficacy testing of the experimental treatment condition is being conducted using a randomized controlled trial (RCT) of 70 veterans with PTSD assigned to receive either RESCUE paired with PE (RESCUE + PE) or community involvement paired with PE (CI + PE). Consistent with the manual guidelines [21], PE consists of up to 12 weekly sessions. Veterans are encouraged to engage in their assigned volunteer activity (either volunteering at the animal shelter or engaging with a community agency, depending on condition) a minimum of once per week. In addition to the baseline assessment, veterans are assessed at mid-treatment (session 5), immediately post treatment (12 weeks), and at the 3-month follow-up (see Fig. 1 below). Clinical outcomes include levels of PTSD, depression, anxiety, and functional status/quality of life. Additionally, after completing treatment, veterans are asked to participate in an individual thematic interview to get their impression of the treatment program overall, their perceptions regarding their current functioning and symptoms relative to the start of treatment, any difficulties/barriers they faced with aspects of the program, and their thoughts about specific components of PE (i.e., imaginal and in vivo) and their volunteer assignment.

### Study aims

1. RESCUE + PE will decrease emotional-numbing symptoms of PTSD relative to the PE control arm (CI + PE) primary outcome
2. RESCUE+PE will be feasible and acceptable to veterans
3. Treatment engagement will be higher for the RESCUE + PE group relative to CI + PE group (i.e., higher treatment attendance rates (primary), homework completion rates, and treatment completion rates)
4. Treatment recovery rates (as measured by PTSD diagnostic status yes/no; decreased PTSD symptom severity; and increased quality of life) will be higher for the RESCUE + PE group compared to the CI + PE group at post treatment and at 3-month follow-up
5. Participants randomized to RESCUE + PE will experience a decrease in PTSD numbing from pre to post treatment relative to those randomized to CI + PI

## Methods/design

### Participant recruitment

Seventy male and female veterans with PTSD are being recruited from a VAMC in the Southeastern United States. Veterans are referred to the study through the PTSD Clinical Team (PCT), general referrals from other VA clinics, posted flyers in approved community locations where veterans are likely to congregate, and word of mouth from other participants or individual providers.

All veterans complete an intake assessment by the PCT prior to study entry. Study staff then follow up with the intake evaluator regarding the outcome of the assessment and whether or not the veteran expressed an interest in being contacted about future opportunities to participate in research. Veterans who appear to meet the criteria for entry into the study (i.e., positive for PTSD, express willingness to engage in EBT for PTSD) and agree to be contacted are telephoned by a study staff member and given a description of the study. If interested in participating, veterans are scheduled for a telephone baseline assessment that includes informed consent. Veterans who live locally and prefer to be seen in person have the option of completing the informed consent procedures and baseline assessment appointment in the PCT clinic. Veterans who receive care via telehealth or prefer to receive their care via telehealth are mailed a consent form and baseline packet prior to their baseline assessment date so that they have a physical copy of the consent to refer to while going over the consent form with a study staff member by telephone. Following the telephone consenting/baseline appointment, participants are asked to mail the signed consent back to the research team using a self-addressed envelope. Once the signed consent form is received and the baseline measures are completed, the veteran is considered eligible for the study.

### Randomization procedures

Once eligibility is established, veterans are assigned 1:1 to one of the two treatment groups by the Project Coordinator using a web-based computer-generated randomization scheme. Once a veteran is randomized and attends the first session, they are entered into the study and included in the intent-to-treat (ITT) analysis. The post-treatment assessors (12- and 3-month assessors) will be blinded to condition. The Principal Investigator (PI) (for clinical oversight/supervision) and Project Coordinator (for randomization/regulatory reasons) will not be blinded to treatment condition. The study therapists likewise will not be blinded because discussion of volunteer assignments is part of the treatment protocol. It is not anticipated that the post-treatment assessors will need to be unblinded as this will be their only point of contact with the study participant. If the blind is accidentally broken, the Clinician Administered PTSD Scale (described below) will be reviewed by another team member for fidelity and evaluated against the PTSD Symptom Checklist (described below) which is self-report.

Inclusion criteria for the study are as follows: (1) *Diagnostic and Statistical Manual for Mental Disorders, version 5* (DSM-V) PTSD diagnosis (via the Clinician Administered PTSD Scale; CAPS [22];) stemming from a duty-related Criterion A event [23]; (2) male or female aged 18 to 64 years.

Exclusion criteria for the study are as follows: (1) presence of an active substance-use disorder that requires medical detoxification; (2) diagnosis of Antisocial Personality Disorder or history of animal cruelty; (3) presence of delirium, dementia, amnestic disorders, or other cognitive disorders and psychotic disorders that would likely interfere with the ability to consent or comply with study procedures; (4) presence of active/uncontrolled bipolar I or II disorder; (5) current use of benzodiazepine medications (if willing, participants are required to taper and cease use under physician supervision, and they must be off the medication(s) for at least 2 weeks prior to enrolling in the study); (6) recent prescription of an SSRI antidepressant medication or a recent change in dosing (participants must be on a consistent dose for at least 2 weeks prior to enrollment and throughout the study); (7) suicidal or homicidal ideation with intent; (8) lack of English language fluency; and (9) presence of a specific phobia related to dogs or any other relevant aversion to dogs (i.e., allergy). Once enrolled, participants will only be discontinued from the study if the study appears to be causing them undue or atypical distress and/or if the patient elects to terminate their study participation.

**Fig. 2:**
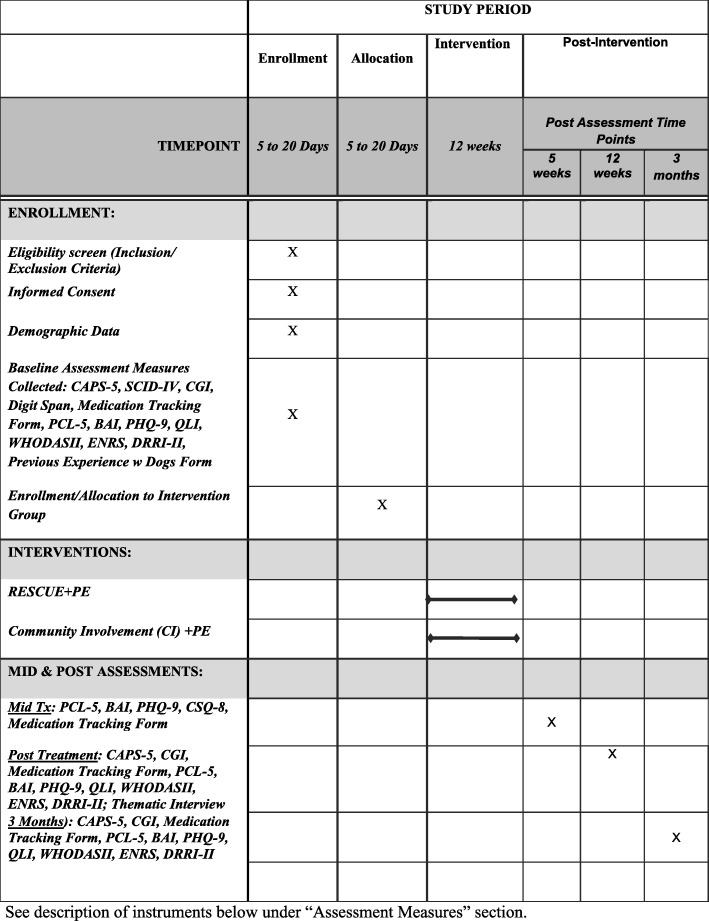
Schedule of assessments and procedures

See description of instruments below under the “Assessment measures” section and Fig. 2 for the study assessments and procedures.

### Randomization and intervention

#### Prolonged Exposure (PE)

All veterans receive individual sessions of Prolonged Exposure therapy for PTSD (PE). PE consists of up to 12 sessions delivered once weekly for 60 to 90 min. Foa’s PE protocol is used, given consensus statements regarding its efficacy for PTSD as well as its wide-scale dissemination [21]. Consistent with the manual guidelines, sessions consist of imaginal (in session) exposure exercises, in-vivo (out-of-session) exposure exercises, review of homework, and relevant processing of in- and out-of-session activities. Study therapists were trained and certified through the VA’s PE certification process and they receive weekly supervision for all cases. Veterans in the RESCUE + PE and CI + PE conditions receive additional psycho-educational materials relevant to either canine shelter volunteering or community agency volunteering, respectively. Discussion regarding experiences with assigned volunteer activities is incorporated into treatment sessions, generally during discussion of homework assignments and homework review. Thus, the control condition matches the experimental condition in relation to both the addition of out-of-session volunteer activities and in-session discussion and processing of those activities.

#### Recovery through Engagement with Shelter Canines, Understanding, and Exposure (RESCUE)

Half of all veterans are randomly assigned to receive RESCUE concomitant with PE. RESCUE volunteer sessions occur once weekly at an area Society for the Prevention of Cruelty to Animals (SPCA) chosen by the veteran and last approximately 90 min. Expectations regarding volunteer assignments are reviewed by study staff prior to the start of treatment and progress or difficulties with the assignments are discussed throughout treatment as described above. Prior to starting their volunteer assignment, participants receive an orientation by SPCA professionals consistent with what is provided to community volunteers. This training includes an orientation to the physical space of the shelter, basic safety and handling education, and discussion of daily tasks involving interaction with the canines that can be accomplished. Participants are limited to working with *non-aggressive* dogs.

#### Community Involvement volunteer condition

Half of all veterans are randomly assigned to participate in Community Involvement concomitant with PE. Veterans are provided with a brief handout listing local community agencies that are actively recruiting or accepting volunteers (e.g., local Young Men’s Christian Association (YMCA) facilities, soup kitchens, housing and community improvement projects, reading partners) but they are also encouraged to choose any agency not listed on the study handout if they prefer. Veterans are specifically instructed to not volunteer at an animal shelter. Veterans are responsible for calling the volunteer agency of their choice and setting up an initial orientation with the facility. They are also asked to engage with the agency at least once per week for the duration of treatment. Study staff are available to help participants coordinate the logistics of initiating and maintaining contact with their chosen community agency as needed. Comparable to the RESCUE condition, study therapists review progress and/or difficulties with the volunteer assignments throughout treatment as part of the homework assignment and review process.

### Assessment measures

Participants are screened for eligibility by the Project Coordinator who has a master’s degree in counseling psychology. This screening/baseline assessment includes informed consent and administration of a battery of measures consisting of the Clinician Administered PTSD Scale-5 (CAPS [22];), the Structured Clinical Interview for DSM-IV Axis I Disorders (SCID-IV) Modules for Mood and Generalized Anxiety [24]; Clinical Global Impressions (CGI [25];); Digit-Span [26]; and a Medication Tracking Measure. Self-Report measures include the Posttraumatic Stress Disorder Checklist (PCL-5 [27];); Beck Anxiety Inventory (BAI [28];); Patient Health Questionnaire (PHQ-9 [29];); Quality of Life Index (QLI [30];); World Health Organization Disability Assessment Schedule II (WHODAS II [31];; Emotional Reactivity and Numbing Scale (ENRS [32];); a study-specific Previous Experience with Dogs Form; Deployment Risk and Resilience Inventory (DRRI-2 [33];). The baseline assessment typically occurs over one to two sessions and veterans are compensated US$50.00 for their time.

Weekly treatment session measures include the PCL-5; Utilization of Treatment Inventory (UTI, weekly [21];), a study-specific Community Involvement Report Form, and a study-specific Violations of Expectancies about Imaginal Exposure Form (VEMIE; sessions 2, 3, and 4). At mid-treatment veterans complete the BAI; PHQ-9; Medication Tracking Measure; and Client Satisfaction Questionnaire – 8 (CSQ-8 [34];).

At post treatment and 3 months, veterans complete the same battery of instruments as those administered during the baseline minus the SCID-IV, Treatment Expectancies Form, and Previous Experience with Dogs Form. The 3-month battery does not include a thematic interview. Mid-treatment and post-treatment follow-up assessments typically occur over one session and veterans are compensated US$50.00 for each assessment completed (i.e., mid-treatment, post-treatment, and 3-month follow-up). Veterans who drop out of treatment prematurely are encouraged to complete the follow-up assessments and are likewise eligible for compensation. All assessments, including the baseline, are conducted by master’s level or above personnel trained in the interview procedures by the PI. Additionally, all assessors participated at the onset of the study in a formal CAPS training provided by a senior clinician in the Charleston PCT clinic. All post assessments are conducted by a study personnel blind to randomization status.

### Thematic interview

As part of the post-treatment assessment, veterans complete a 30–45-min individual thematic interview designed to get their impression of the treatment program overall, their perceptions regarding their current functioning and symptoms relative to the start of treatment, any difficulties/barriers they faced with any aspect of the program, and their thoughts about specific components of PE (i.e., imaginal and in vivo) and their volunteer assignment. Aside from gathering information to potentially improve the program moving forward, it is anticipated that the thematic interview will provide a better understanding of if, and how, HAI and the control volunteer assignment impacted veterans with regard to their symptoms and/or quality of life.

### Study/methods monitoring

This study was approved by the Medical University of SC Institutional Review Board (IRB) (Pro#00053520), the Charleston VA Research and Development Office; and the Department of Defense Human Research Protection Office. All participants receive verbal information about the study and a written and signed informed consent document is obtained for study purposes. Quarterly progress reports are submitted to the DoD and annual audits of the study are conducted by the MUSC IRB. Any adverse events are required to be reported to the IRB within 24 h and the study PI must respond in writing to the IRB regarding any needed change in procedures as applicable. MUSC IRB, the Charleston VA Research Office, and the Department of Defense Human Research Protection Office will monitor adverse events related to the study with regard to frequency, severity, and causality and will use these factors to determine whether the study should be terminated given the risk to benefit ratio. Any changes to the study protocol must be approved by MUSC IRB and The Department of Defense Human Research Protection Office; and updates to the ClinicalTrials.gov registry are made as applicable. A data monitoring committee was not established because the clinical trial is relatively small and poses minimal risk beyond usual care for the treatment of PTSD (i.e., usual care for PTSD is Prolonged Exposure). Although minimal risks with participation are anticipated, the Charleston VAMC will cover non-negligent harm associated with study participation and will provide alternative and/or additional medical services that may arise as consequence of study participation as part of the Veterans Health Administration.

### Data management

Data is compiled using codes in lieu of personal identifiers, and access to study data is limited to research personnel. Development of, and security oversight for, the electronic database for this study is performed by study personnel using a secure, web-based application to support data capture. The application provides: (1) an intuitive interface for data entry (with data validation); (2) audit trails for tracking data manipulation and export procedures; (3) automated export procedures for seamless data downloads to common statistical packages (SPSS, SAS, Stata); (4) procedures for importing data from external sources; and (5) advanced features, such as branching logic and calculated fields. Only de-identified data is entered into the electronic database. The data-entry management system is accessible from the Charleston VAMC. Although no Protected Health Information (PHI) is entered into the database, data system security is ensured by implementing multiple-layered firewalls and a network intrusion prevention system for identifying and blocking malicious network activity in real time. A hard-copy study log linking patient names with study ID numbers is kept in a locked cabinet in a secure room at the Charleston VAMC, and access to this log is limited to only approved few study personnel. The PI and Project Coordinator will have direct access to the dataset (interim and final) and will provide access to other Co-Investigators (Co-Is) on the study if requested once the dataset is finalized. A procedure for interim analyses was not included as part of the data management plan given the low anticipated risk to participants.

### Sample size calculation and power analyses

For the continuous longitudinal clinical, functional, and process outcomes for Primary Aims, with 35 subjects randomized to each intervention group, we will have 85% power to detect at least a 0.3 standardized (sd units) effect size between the two intervention groups assuming three time points, and an intra-class correlation no greater than 0.5; level of significance ≤ 0.05, two-tailed. A standardized effect size of 0.35 sd (Cohen’s *d*, “small” effect) is equivalent to a raw effect size of 2.8 raw scale units for the PCL (assuming pooled sd for post-treatment PCL scores is 8.1, from preliminary data). All participants randomized to condition and having completed a baseline assessment will be included in ITT analyses.

### Analytic strategy

Data screening for violations of proposed statistical methods assumptions (normality of endogenous variables, linear relations among variables, no outliers, limited missing data/appropriate method for handling missing data) will include histograms, bivariate correlations, Q-Q plots, and other diagnostic methods. Processes associated with subject attrition will be examined to determine whether a missing-at-random assumption is defensible. Data will be analyzed to determine whether missing values are correlated with any baseline values and all relevant psychometric variables will be examined between groups at the baseline point of measurement to ensure adequate randomization. Given limitations of last-observation-carried-forward (LOCF), hierarchical linear models (HLM)/mixed models will be used for ITT analyses of primary and secondary outcome variables. Statistically significant treatment-related changes will be qualified with effect-size estimation and 95% confidence interval estimation.

### Feasibility/acceptability

Feasibility and acceptability will be assessed via rates and levels of recruitment, retention, and PE homework compliance rates (engagement), feedback from therapists and outside experts, perceived treatment satisfaction, and through the thematic interview data.

### Treatment effects

Treatment effects will be examined via changes on continuous measures of PTSD numbing symptoms from the CAPS-5, PCL-5, and ERNS as well as by changes on continuous CAPS-5 and PCL-5 total scores, other associated mental health outcomes (e.g., BAI, PHQ-9), and functional status/quality of life (e.g., WHODAS-II; QLI). CAPS-5 and PCL-5 emotional-numbing items will be analyzed separately based on factor analytic findings and the current hypothesis that RESCUE will specifically decrease emotional-numbing symptoms.

To evaluate pre-post treatment continuous outcome measures, HLM/mixed models will be estimated with randomized group entered as a level-2 fixed effect. Effect sizes and 95% confidence intervals will be employed in a manner consistent with accepted norms [35]. For multiple point measurement related to self-report measures, estimates of slope will also be tested. For dichotomous outcomes, rates will be assessed by chi-square comparison of between-group percentages. Diagnostic improvements will be examined by computing the percentage of treated participants who no longer meet PTSD criteria at each follow-up. Outcomes will also be evaluated using CGI-I scores at all post-treatment follow-ups (i.e., percentage of veterans receiving a CGI-I rating of 1 or 2).

### Dissemination of study findings

The study team will conduct dissemination activities throughout the project period— both locally within the Charleston VA, in informal presentations to other southeastern VA hospitals, and across the country at professional conferences. We will also use our own core and extended collaborative relationships to distribute information. Specifically, dissemination activities will include (1) authorship of descriptive materials that outline the study and its objectives in the form of white papers and brochures; (2) publication of the study results in professional and non-professional outlets, such as psychiatric and medical journals; (3) presentations and lectures in the form of graduate courses on treatment outcome research, seminars on innovations in treatment, invited grand rounds at our respective and other Universities, meetings describing project results; (4) active, ongoing evaluation of these activities; and (5) distribution of summary data to relevant stakeholders, including VA National Centers for PTSD (i.e., the PTSD Mentoring Program and the Practice Based Implementation Network), DoD, and other VAMCs.

The study PI will record and track all dissemination activities and direct project Co-Is and other study personnel to increase or widen the scope and frequency of these activities when appropriate. She will also actively participate in the majority of these activities herself. The PI will coordinate the data analysis for the project, relevant manuscript submissions, presentations, mailings and other forms of distribution, publicity, etc. The PI and Co-Is will author all manuscripts related to the project (i.e., no professional writers will be used). The PI will also guide dissemination efforts through the national programs described above. No more than 3 years after the collection of the 3-month post-assessment interviews, the PI will provide a de-identified dataset to an appropriate data archive for sharing purposes. The study protocol will be available upon request within 6 months of the study end date.

## Discussion

Despite systemic efforts by the DoD and VA to ensure that EBTs are available to all veterans with PTSD, many veterans are not initiating or completing treatment. This is a significant issue given that PTSD does not typically remit over time and is associated with significant impairment across a range of psychosocial indices. Given the increased availability of EBTs, and the well documented and robust effect sizes for patients who complete PE, more attention should be given to testing novel methods for improving treatment engagement and retention rather than further improving outcomes among those who comply with PE treatment guidelines. As with other psychiatric disorders (e.g., depression), the symptoms that pose as a barrier to treatment engagement should be targeted directly to improve treatment initiation, engagement, and completion [36]. In this effort, the current clinical trial focuses on addressing the emotionally numbing symptoms of PTSD using a HAI model.

In addition to being part of the symptom profile of PTSD, emotional numbing can serve as a barrier to care through its heightened association with apathy, low motivation, and feeling disconnected from others [10, 14]. Unfortunately, current EBTs do not target emotional numbing directly. Thus, although emotional-numbing symptoms can decrease with treatment of a sufficient duration, targeting them proactively and effectively in the early stages of treatment is likely to improve overall treatment retention and subsequent clinical improvement. Based on the broader HAI literature, RESCUE (the experimental condition) was designed explicitly as an adjunctive therapy to augment extant EBTs by decreasing emotional-numbing symptoms, increasing veteran “buy in,” facilitating avenues for effective EBT psycho-education, and increasing supportive and therapeutic in-vivo exposure and commensurate behavioral activation, thereby improving PTSD EBT engagement and completion rates.

A strength of the proposed project is that it is designed to target the significant number of veterans and active duty personnel with PTSD who are at risk for refusing or dropping out of EBTs [4, 5, 7, 37]. Additionally, RESCUE incorporates HAI into the chronological treatment progression of PE by taking advantage of an already existing and disseminated infrastructure related to (1) volunteering at an SPCA to socialize shelter dogs for eventual adoption and (2) standard PTSD treatment guidelines (i.e., in-vivo exposures) as mandated by the Uniform Mental Health Services Handbook (VHA HANDBOOK 1160.01). By taking advantage of the availability of SPCA shelters nationally as well as existing service delivery models for PTSD, RESCUE can easily be scaled up for wide-scale implementation and dissemination. Further, the theory-driven mixed quantitative/qualitative methods design of this trial will allow for a better understanding of how HAI may be beneficial for individuals with PTSD as well as provide information on how to further improve the treatment program moving forward. Altogether, this study should provide timely information regarding incorporating HAI into EBTs for PTSD.

A significant contribution of the current study is in relation to specifying an ecologically valid, active, and well-matched control condition designed to determine animal-specific or HAI-specific effects. Additionally, much of the extant literature to date has focused on the beneficial health effects of HAI through long-term pet ownership rather than the effects of specific interventions. Finally, reviews of HAI specifically related to therapeutic interventions or specific clinical populations suggest an overall lack of methodological rigor [16, 18–20] which is a considerable barrier to progress in the field. The current study team noted these trends when designing the trial and decidedly erred on the conservative side in relation to specifying the control group. The control group is well matched in and outside of treatment sessions and participants are randomly assigned to conditions. Additionally, the experimental condition is not related to receiving additional services or a service dog, and therapy is provided using an ecologically valid treatment setting and referral stream.

Despite the above study strengths, a few limitations merit note. First, it was not practical or possible to blind the study therapists to condition given the treatment context which necessitated discussion of the volunteer assignment during sessions. However, the current study team’s research focus and experience is related to treating PTSD in VA contexts using exposure-based interventions, and not to HAI. Thus, the potential for implicit bias toward the experimental condition is somewhat mitigated. Second, given that veterans in the CI + PE group can self-select their volunteer activity, factors related to preference, comfort, and scheduling ease (i.e., ease of choosing a volunteer assignment close to home) could obscure potential gains related to RESCUE. The use of both quantitative (i.e., tracking of attendance and discussion of volunteer successes and challenges as part of therapy) and qualitative data (i.e., thematic interviews), however, will allow for a better understanding of the impact of these factors on outcomes. Finally, as is often the case with clinical trials, the current study design is geared to provide more easily interpretable effects given a positive outcome rather than a negative outcome. Although this is important for HAI research at this stage, negative findings will be somewhat more difficult to interpret and would not rule out the potential for other more intense, or more active HAI augmentations to EBTs.

## Implications and conclusions

PTSD is one of the most prevalent mental health disorders among post-deployed veterans [38]. Despite the wide-scale availability of EBTs for PTSD within DoD and VA health care settings, however, a significant number of veterans and active duty personnel fail to initiate treatment or drop out of treatment prematurely before experiencing meaningful symptom relief. The current study is designed to address these barriers to care through RESCUE, a novel adjunct to PE based on findings regarding emotional numbing and HAI therapeutic models. If proven efficacious, RESCUE has the potential to yield significant and meaningful cost savings at the individual level with regard to suffering and lost work productivity as well as at the systems level with regard to disability and health care utilization patterns.

## Supplementary information

**Additional file 1.** Standard Protocol Items: Recommendations for Interventional Trials (SPIRIT) 2013 Checklist.

## Data Availability

The data that support the findings of this study are available from the corresponding author upon reasonable request.

## Abbreviations

CI: Community Involvement
DoD: Department of Defense
EBT: Evidence-based treatment
HAI: Human-animal interaction
OIF/OEF: Operation Iraqi Freedom; Operation Enduring Freedom
PCT: Posttraumatic Stress Disorder Clinic
PE: Prolonged Exposure
PTSD: Posttraumatic stress disorder
RCT: Randomized controlled trial
SPCA: Society for the Prevention of Cruelty to Animals
VAMC: Veterans Affairs Medical Center
YMCA: *Young Men’s Christian Association*
